# The checklist checkup & checklist fatigue – Say what? How does it impact clinical perfusion practice

**DOI:** 10.1051/ject/2023046

**Published:** 2024-03-15

**Authors:** Bharat Datt

**Affiliations:** President – CanAmerica Cardiopulmonary LLC 12030 Sawgrass Reserve Blvd Orlando FL 32824 USA

Checklists comprise an important part of the perfusionist’s clinical practice. How many times have we forgotten important steps before setting up or priming, especially when we are in a hurry? The Agency for Healthcare Safety and Quality (AHRQ) defines a checklist as “an algorithmic listing of actions to be performed in a given clinical setting, the goal being to ensure that no step will be forgotten”. Checklists are said to improve quality of care- E.g.: Surgeons experienced 50% fewer positioning errors when dealing with laparoscopic equipment when they used checklists [1].

**Figure 1:**
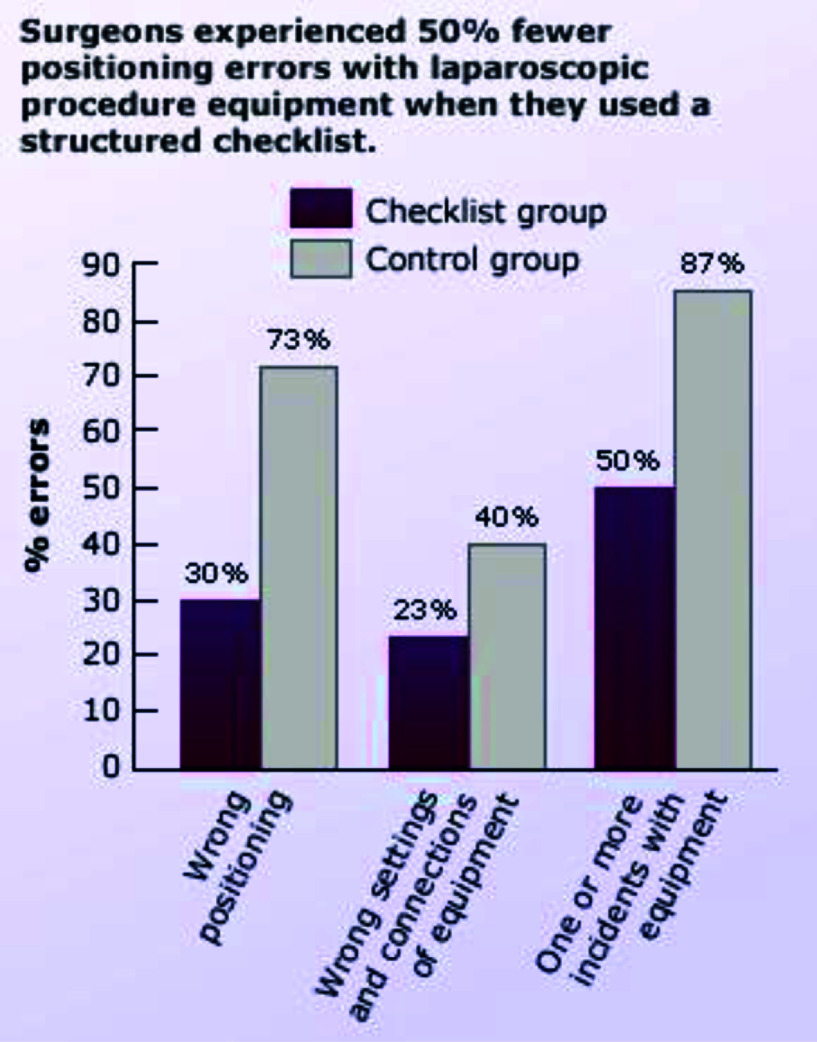
Structured checklists help reduce errors.

**Figure 2:**
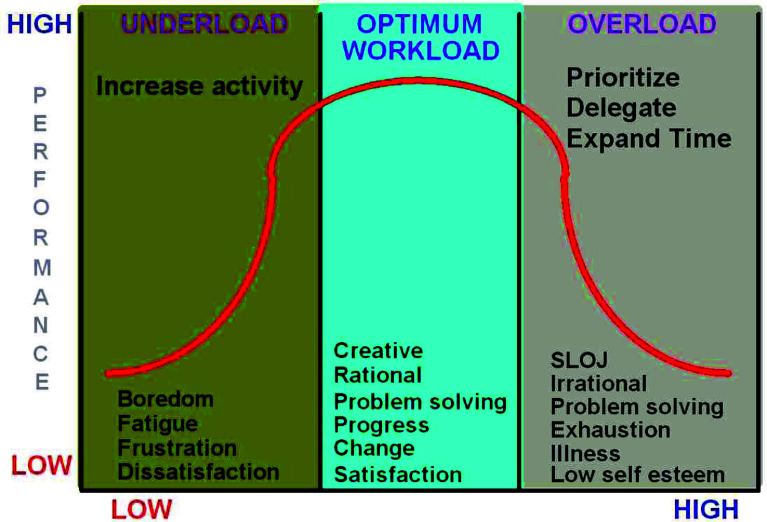
Optimum workload is important for safety and quality. Credit: https://www.cfidarren.com/crmworkload.htm.

We can all agree that checklists have been a lifesaver as we progress in our perfusion practice journey.

Or is it? Can we take steps to improve the utilization of checklists in our field?

Being self-aware has been quoted to be a critical leadership skill [2]. But what does it have to do with checklists? I am self-aware enough to acknowledge that there are times when I do not pay proper attention to the checklist- Especially when I have time. I have a tendency to memorize my checklist, which is not a good idea. The Federal Aviation Authority (FAA) fails a pilot in his or her test if a physical checklist is not used and disallows checklists by rote. Memory is not considered an acceptable substitute for a physical checklist [3].

There is ample evidence to suggest that there are drawbacks to the utilization of checklists in healthcare. The main barriers to implementation are listed as below [4]

(a) Staff attitudes
(b) Hierarchies
(c) Poor design
(d) Duplication with other worklists
(e) Work overload
(f) Cultural barriers.

Post COVID pandemic we are all familiar with work overload.

However, I have definitely caught myself ticking off the checklist without paying direct attention to the tasks listed on the checklist. It is my opinion that the reason of course is my attitude, but also poor design. Why do we need a “restock the cart” in our checklists? Checklists should be comprehensive but not too detailed. Otherwise, it can lead to checklist fatigue. Yes! It’s a real thing [5]. I have also been told by some of my colleagues that checklists need to be detailed in clinical rotation sites, because of students. My rejoinder would be that the school can have a checklist specifically for students.

To conclude, it’s my opinion that the answer lies in standardization- Just as we have done in a plethora of clinical activities, safety devices and safety training. The American Society of Extra-Corporeal Technology (AmSECT) has a template for checklists. Institution-specific add-ons can be made, but I believe standardization and optimization are key. Standardization will enable perfusionists to retain key checklist parameters while eliminating the fluff. Education about paying attention to checklists without rote memorization should be a key part of student training and education. At the very least, the perfusion community can benefit from a conversation around the checklist checkup.

## References

[1] Verdaasdonk EG, Stassen LP, Hoffman WF, et al. Can a structured checklist prevent problems with laparoscopic equipment? Surg Endosc. 2008;22(10):2238–2243. 18597142 10.1007/s00464-008-0029-3

[2] Hougard R, Carter J, Afton M. Self-awareness can help leaders more than an MBA. Harvard Business Review. 2018. Available at: https://hbr.org/2018/01/self-awareness-can-help-leaders-more-than-an-mba-can.

[3] McClellan M. Checklists vs memory items. Air Facts. 2021. Available at https://airfactsjournal.com/2021/08/checklist-vs-memory-items; https://www.cfidarren.com/crmworkload.htm.

[4] Concha-Torre A, Diaz Alonso Y, Alvarez Blanco S, et al. The checklist’s: A help or a hassle. An Pediatr (Barc). 2020;93(2):135.e1–135.e10. 10.1016/j.anpedi.2020.05.006 32591318

[5] Grigg E Smarter clinical checklists: How to minimize checklists fatigue and maximize clinician performance. Anesth Analg. 2015;121(2):570–573. 26197378 10.1213/ANE.0000000000000352

